# The Dominance of Liking: Uncovering Dyadic and Reputational Effects of Peer and Perceived Teacher Likes and Dislikes on Friendship Dynamics Among Chinese Adolescents

**DOI:** 10.1007/s10964-024-02104-5

**Published:** 2024-10-30

**Authors:** Xingna Qin, Lydia Laninga-Wijnen, Christian Steglich, Yunyun Zhang, Ping Ren, René Veenstra

**Affiliations:** 1 Department of Sociology, University of Groningen, Groningen, The Netherlands; 2 Collaborative Innovation Center of Assessment toward Basic Education Quality, Beijing Normal University, Beijing, China; 3 Department of Behavioral Sciences and Philosophy, University of Turku, Turku, Finland; 4 Institute for Analytical Sociology, Linköping University, Linköping, Sweden; 5 State Key Laboratory of Cognitive Neuroscience and Learning, Beijing Normal University, Beijing, China

**Keywords:** Adolescents, Teacher liking and disliking, Peer liking and disliking, Friendship network

## Abstract

While previous research suggests that peer and teacher preferences are linked to adolescents’ peer relationships, the specific impact of peer and teacher (dis)liking on adolescents’ friendship networks is not fully understood. This study used longitudinal social network analysis to examine how peer (dis)liking and perceptions of teacher (dis)liking predicted friendship selection among Chinese adolescents. Questionnaires were administered to a sample of 2566 students (48.3% boys, *M*_age_ = 13.94, SD_age_ = 0.60 at Time 1) in central China in 2015 and 2016. Results for peer (dis)liking revealed that Chinese students tended to befriend peers they liked (dyadic perception), to befriend peers widely liked (reputational perception), and to avoid peers widely disliked (reputational perception). Regarding teacher (dis)liking, Chinese students tended to befriend peers they believed their teachers liked (dyadic perception) and avoid those widely perceived as liked by teachers (reputational perception). Interestingly, students who were widely perceived as liked by teachers tended to befriend peers whom they believed teachers disliked. Perceived teacher disliking had a limited effect on friendship selection at both the dyadic and reputational levels. These findings suggest that peer liking and perceived teacher liking relate to friendship formation among Chinese adolescents, but that a reputation as a teachers’ pet may hinder their friendships.

## Introduction

Friendships play a critical role in the social and emotional development of adolescents, serving as resources for psychosocial and academic support (Parker & Asher, 1993; Stanton-Salazar & Spina, 2005). The absence of friendships can lead to feelings of loneliness, increased aversion to school, and increased vulnerability to peer victimization (Bagwell & Bukowski, 2018). However, not all peer interactions lead to friendships; adolescents’ preferences or repulsions, such as liking and disliking, are assumed to drive friendship formation (Beazidou & Botsoglou, 2016). Teachers can also influence these dynamics by serving as social referents, with students inferring peers’ characteristics based on teacher-student interactions (Hughes et al., 2001). Despite the shared social environment between students and teachers, research has focused primarily on peer dynamics, leaving a limited understanding of how perceptions of both peer and teacher preferences affect friendship networks. This study addresses this gap by using longitudinal social network analysis to examine how adolescents’ own liking and disliking and their perceptions of teachers’ liking and disliking influence friendship dynamics.

### Peer (Dis)liking: Dyadic Affection and Reputational Status on Friendship Selection

Peer liking and disliking refer to the positive or negative feelings and attitudes that adolescents have toward each other (Buhs & Ladd, 2001; Card, 2010; Hughes & Im, 2016). These preferences are typically assessed through sociometric procedures, such as peer nominations or ratings (Hughes & Im, 2016). Peer nominations provide at least two types of information. First, they create a network of dyadic (dis)liking nominations, allowing researchers to explore who likes whom and how these dyadic relations affect certain outcomes. Second, peer nominations enable researchers to examine reputations for (dis)liking by counting the number of times an individual is nominated as liked or disliked by others (Rubin et al., 2006). Dyadic and reputational peer (dis)liking are two distinct but related constructs (Palacios et al., 2022). The former refers to the mutual affection or positive relationship between two individuals (Buhs & Ladd, 2001), and the latter refers to the overall perception of students within the larger peer group, which encompasses the collective sentiment or reputation students hold among their classmates (Hughes & Im, 2016). This distinction highlights the need to explore both perspectives in understanding friendship dynamics. However, existing research has predominantly focused on reputational (dis)liking, leaving a gap in understanding how dyadic (dis)liking impacts friendship dynamics.

From a reputational perspective, students’ reputations for being (dis)liked by peers may determine whether or not they are seen as attractive friendship partners. Students who are well-liked by others are generally perceived as friendly, helpful, cooperative, and good leaders (Rubin et al., 2006) and less physically aggressive and more prosocial (Shin, 2017), making them attractive as potential friends. In addition, social preference may be an indicator of a student’s social status in the peer group, which adolescents strive to improve. Social status becomes a highly salient determinant of friendship formation among adolescents (Shin, 2017). Befriending students with high social status can be an effective way to “bask in reflected glory” and improve an individual’s social status (Dijkstra et al., 2013). Conversely, students who are disliked by many of their peers may be seen as unattractive friendship partners, for example, because these peers may be at increased risk of victimization and thus jeopardize the social status of their friends (Twenge et al., 2001). As a result, students who are well-liked by their peers are assumed to be attractive to befriend, while those who are widely disliked by their peers are more likely to be rejected and typically avoided (Ryan & Shin, 2018).

While much research has focused on reputational peer (dis)liking and its impact on friendship selection, less is known about the role of dyadic (dis)liking in these processes. From a dyadic perspective, students tend to form friendships with those they like. Research indicates a positive correlation between liking nominations and friendship nominations in Canadian and American elementary schools (Guimond et al., 2022). Elementary school students typically rate their best friends as the peers they liked the most (Yugar & Shapiro, 2001). Furthermore, a social network study suggests that children defend classmates with whom they like (or friends) and who like them, but do not defend classmates who they dislike and who dislike them (Rambaran et al., 2022). In terms of peer disliking, research on the co-evolution of peer disliking and friendship is scarce. Notably, a recent longitudinal study found that students were more likely to bully peers they disliked (Kisfalusi et al., 2022). Given that bullying and victimization typically do not occur within established friendships, this suggests a potential negative relation between peer disliking and friendship selection.

### Teacher (Dis)Liking: Social Reference in Friendship Selection

Although adolescents typically seek and develop friendships based on their own interests, personalities, and social dynamics, teachers may also play a role in friendship selection. Consistent with ecological models of development (Bronfenbrenner & Morris, 2006), interactions with both teachers and peers in the classroom context are considered proximal influences on adolescents’ social, emotional, and academic development (Hughes, 2012). However, as adolescents become more attuned to their peers, the question arises as to whether teachers still influence friendship dynamics or whether they are largely on the sidelines.

Teachers can shape adolescents’ friendship networks through their own relationships with the students, serving as a “blueprint” or social reference for affective evaluations of peers (Endedijk et al., 2022; Hughes et al., 2001; McAuliffe et al., 2009). Similar to peer (dis)liking, students’ beliefs about teacher (dis)liking may also affect friendship dynamics in two ways: dyadic and reputational. Dyadic perceived teacher (dis)liking reflects individual observations of teacher-student interactions, capturing personal perceptions of the teacher’s attitude toward that particular student. Reputational teacher (dis)liking is driven by the frequency with which an individual is nominated by others as liked or disliked by teachers, representing the collective sentiment of the group about the teacher’s attitude toward a student (Hughes & Im, 2016). Discrepancies between these individual and collective perceptions may arise from a variety of factors, including differences in personal interactions, differences in interpreting teacher behavior, or potential misperceptions or biases about the teachers’ feelings. Both dyadic and reputational teacher (dis)liking reflect students’ perceptions of teacher-student relationships and may guide students’ friendship selection processes in the classroom.

From a reputational perspective, student who are perceived by many others to be liked by teachers are often seen as accepted and attractive by classmates. For example, a longitudinal study of 5th and 6th graders found that students who are liked by their teachers tend to be included by their peers and then to perform successfully in school (Sette et al., 2020). While previous studies have found that being liked by teachers promotes positive outcomes, such as positive peer relationships and high academic achievement (e.g., Hendrickx et al., 2017a; Hughes et al., 2001; Sette et al., 2020), being liked by teachers can also have negative consequences for adolescents, such as being labeled a “teacher’s pet” (Babad, 2009). When students believe that a teacher favors a student, it can lead to dislike or rejection of the student (Babad, 2009). This teacher-pet phenomenon has been observed in both Western and Chinese contexts. For example, in the United States, positive teacher behavior was negatively associated with peer liking among elementary school students (McAuliffe et al., 2009). In a study of elementary school students in Hong Kong, students who were liked by their teachers but not by their peers were identified as “teacher pets” and exhibited withdrawal behaviors (Lu et al., 2015). Despite these implications, the teacher-pet phenomenon has been surprisingly understudied, particularly regarding its influence on friendship dynamics.

However, students who are perceived as disliked by teachers may also face social challenges. Whereas previous studies have largely focused on the effects of positive teacher-student relationships, few studies have examined how teacher disliking impacts peer relationships, particularly in friendship selection. One of the few studies in this area, using video vignettes, found that both a student reputation and teacher feedback significantly impacted how young children perceived their peers, with negative teacher feedback being especially influential (White & Kistner, 1992). Thus, students with a reputation for being disliked by their teachers may be less likely to be selected as friends, yet this area remains surprisingly understudied, particularly in terms of its implications for friendship selection.

From a dyadic perspective, perceived teacher (dis)liking can model how students evaluate peers and influence friendship selection (Hendrickx et al., 2017a). Students are likely to adjust their views of classmates based on whether they perceive teachers like or dislike them, a process known as social referencing (Farmer et al., 2011). For example, a social network study found that students tend to like classmates whom they believe the teacher likes and dislike those whom they believe the teacher dislikes (Hendrickx et al., 2017a).

Moreover, the extent to which these social referencing effects take place, may depend on the *quality of the student-teacher relationship*. A positive teacher-student relationship can lead students to conform to their teacher’s preferences or repulsions, making the teacher a relevant and credible model for friendship selection. Conversely, a negative relationship may lead students to resist or disregard the teacher’s preferences. This aligns with social balance theory (Heider, 1946), which posits that consistency in positive and negative social relationships drives attitudes. The current study considers students’ reputations for teacher (dis)liking as reflective of the overall quality of the teacher-student relationship. A reputation for being liked by the teacher likely indicates a high-quality relationship, as it is something visible and recognizable to many students. This study will examine whether students’ reputations for teacher (dis)liking moderates the link between dyadic perceived teacher (dis)liking and friendship selection. It is likely that students who have a reputation to be liked by teachers (and thus, have a good relationship with their teacher) are more likely to follow the teacher’s affective attitudes by befriending peers perceived to be liked by the teacher and avoiding those perceived to be disliked. When students have a reputation for being disliked by the teacher, the impact of perceived dyadic teacher (dis)liking on their friendship selection may differ. However, no previous studies have examined the moderating role of a negative teacher-student relationship. Therefore, this study aims to extend prior research by exploring whether the effects of perceived teacher (dis)liking on students’ friendships selection are moderated by the students’ reputations for being liked or disliked by teachers.

### Peers and Teachers in the Chinese Context

Chinese adolescents growing up in a collectivist society are deeply influenced by Confucian principles, which significantly shape their attitudes and behaviors in the classroom. First, the collectivist culture emphasizes interpersonal harmony, humility, and treating others with respect (Gabrenya & Hwang, 1996). As a result, Chinese students often perceive themselves as closely connected to others and therefore may be less likely to openly express dislike or engage in direct rejection. Second, traditional Confucian values in China emphasize the importance of supporting education and respecting teachers (Jia et al., 2009). This cultural context creates an expectation that students will demonstrate obedience, humility, and deference to their teachers. Such expectations, in turn, can potentially influence students to adopt their teachers’ (dis)favorable attitudes toward others. For example, given the high value placed on academic success in China, teachers may like high-achieving students, reward them with leadership positions, or hold them up as role models. As a result, these high-achieving students are likely to be liked by most, if not all, of their peers. Despite these influential factors, comprehensive empirical studies of the effects of both teacher and peer preferences on adolescent friendship dynamics in China are lacking. Therefore, it is important to examine how Chinese teachers’ attitudes influence students’ friendship selection.

## Current Study

Despite the importance of friendship among adolescents, the mechanisms underlying friendship formation remain underexplored, particularly regarding how peer and perceived teacher (dis)liking at both the dyadic and reputational levels in non-Western contexts such as China. This study aims to address these gaps by examining the influence of both peer and teacher preferences and repulsion, from dyadic and reputational perspectives, on friendship dynamics among Chinese middle school students, using longitudinal social network analysis. For *peer (dis)liking*, from a dyadic perspective, it is hypothesized that when students liked another peer in the classroom, they would be more likely to befriend that particular peer (Hypothesis 1a), and when students disliked a peer, they would be less likely to befriend that peer (Hypothesis 1b). For reputation, it was expected that being liked by many other peers (i.e., peer liking reputation) would lead to receiving more friendship nominations (Hypothesis 2a), whereas being disliked by peers (i.e., peer disliking reputation) would lead to receiving fewer friendship nominations (Hypothesis 2b). For teacher (dis)liking, from a dyadic perspective, it was expected that if students believed that teachers liked a particular peer in the classroom, they would be more likely to befriend that peer (Hypothesis 3a), whereas if students perceived that teachers disliked a particular peer, they would avoid befriending that peer (Hypothesis 3b). This study will explore how perceived teacher’s liking reputation influences students’ friendship dynamics, as it is not yet known whether a perceived teacher’s liking reputation leads to a “halo” effect or instead leads classmates to view a student as a teacher’s pet. Regarding teacher disliking reputation, it was expected that students with a teacher disliking reputation would receive fewer friendship nominations (Hypothesis 4). Furthermore, this study examined whether the direct effects of dyadic teacher (dis)liking on adolescents’ friendship selection dynamics could be moderated by students’ own teacher liking and disliking reputations. It is expected that students’ teacher liking reputations would amplify teachers’ modeling role, leading them to befriend peers whom they perceived to be liked by teachers (Hypothesis 5a) and avoid peers whom they perceived to be disliked by teachers (Hypothesis 5b). In addition, this study will explore the moderating role of teacher disliking reputation in the link between dyadic teacher (dis)liking and friendship formation.

## Methods

### Participants and Procedures

Data for this study were collected as part of a longitudinal study conducted in four waves. The study was conducted in seven randomly selected public general middle schools in central China, with a six-month interval between each wave. A total of 47 classrooms were included, with 13 classrooms in two urban schools, 15 classrooms in one suburban school, and 19 classrooms in four rural schools. Permission to conduct the study was obtained from each school, and informed consent was obtained from both students and parents (or guardians) at each wave.

The surveys central to the current study were administered at two time points: the end of the first semester of eighth grade (December 2015, T1) and the end of the second semester of eighth grade (June 2016, T2) in 47 Chinese classrooms. At T1, 2658 adolescents participated. At T2, 27 students joined, whereas 119 students in two classrooms moved to other classrooms and were excluded from the analysis. Finally, *n* = 2566 students from 45 classrooms participated at two time points (48.3% boys, *M* = 13.94 ± 0.60 years at T1). Each classroom had between 45 and 67 students (*M* = 57). Missing rates at T1 and T2 were 2.1% and 6.0%, respectively, due to students being absent from school on the day of testing or voluntarily withdrawing from the study.

The data collection procedure involved students filling out paper questionnaires during regular class time. Trained undergraduate or graduate students supervised the process. The classroom teacher was also present to answer any questions and to ensure that students completed the questionnaire without distraction.

### Measures

The peer nomination procedures assessed friendships, peer liking and disliking, and perceptions of teacher liking and disliking. Participants were asked to review a list of all classmates’ names and nominate up to five classmates per peer nomination.

#### Friendship Networks at T1 and T2

At each measurement occasion, students were asked to nominate best friends by answering the question: “Which classmates are your best friends?” The nominations were then transformed into adjacency matrices for each classroom at each assessment. In these matrices, nominations were coded as 1, indicating a tie from one student (in a row) to the other (in a column), while non-nominations were coded as 0, indicating no connection. Missing data due to non-response were handled using standard RSiena procedures: “last observation carry forward” (Ripley et al., 2023). Participants who joined and left the classroom network between two time points were considered structural zeros.

#### Peer (dis)liking at T1

Students were asked to nominate who they liked most and who they liked least. For *dyadic* peer (dis)liking, adjacency matrices were constructed for each classroom at T1. In these matrices, a value of 0 represented the absence of a nomination between two students and 1 represented the presence. For *reputational perceptions of peer (dis)liking*, this study used the proportion scores of peer liking or disliking within classrooms ((the number of nominations received -1) / classroom size).

#### Perceived Teacher (dis)liking T1

Students were asked to indicate their perceptions of teachers’ liking or disliking of their classmates by nominating “Which classmates are liked most by the teachers?” and “Which classmates are liked least by the teachers?”. The questions did not refer to a specific teacher, but to teachers involved in the class in general. Similar to peer liking and disliking, this study constructed adjacency matrices for the *dyadic effect* of teacher liking and disliking. *Reputational perceptions of teacher (dis)liking* were calculated by multiplying the proportion of teacher liking or disliking within classrooms (the number of nominations received -1) / classroom size.

#### Gender

Gender was coded as 0 = boys and 1 = girls.

### Analytical Strategy

#### RSiena

The analyses in this study were conducted using longitudinal social network analyses known as stochastic actor-oriented models (SAOMs) implemented in RSiena (Simulation Investigation for Empirical Network Analysis software package in R, version 1.2–12 in R 3.5.1). The RSiena program allows the estimation of the effects of dyadic or reputational perceptions of both teacher and peer liking and disliking on the friendship network, while controlling for structural network effects (e.g., transitivity) and individual student covariates (e.g., gender) (Ripley et al., 2023).

In this study, the following effects were included in each model: rate effects, network structure effects, dyadic effects, and covariate effects. By including these different types of effects in the analysis, it helps to gain a comprehensive understanding of the factors that shape the evolution of the friendship network over time.

Rate effects were included to model the basic tendencies of actors to form and maintain friendships. Network structure effects were included based on a previous study (Palacios et al., 2022): density, reciprocity, transitivity, outdegree popularity and outdegree activity, outdegree activity, indegree popularity, and outdegree. Appendix 1 contains information on these effects.

The dyadic effects were added to examine the effects of dyadic peer (dis)liking ties and dyadic teacher (dis)liking ties on friendship dynamics. The analysis of dyadic peer (dis)liking examined the extent to which changes in ego liking or disliking led to changes in ego friendship (Hypothesis 1a and Hypothesis 1b). For dyadic teacher (dis)liking, it examined the extent to which ego thinking the teacher likes or dislikes alter leads to ego befriending alter (Hypothesis 3a and Hypothesis 3b). In line with previous research (Babad, 2009; Hendrickx et al., 2017a), teacher (dis)liking refers to students’ perceptions that their teacher likes or dislikes peers, which is a social cognitive tie in the sense that students identify peers whom they believe their teacher likes or dislikes.

The alter of covariate effects were added to examine whether students having a peer (dis)liking reputation or a teacher (dis)liking reputation influenced the friendship nominations they received. For peer (dis)liking reputations, this study examined the extent to which students being liked or disliked by many peers influences their received friendship nominations (Hypothesis 2a and Hypothesis 2b). For teacher (dis)liking reputations, this study examined the extent to which students who perceived by many others to be (dis)liked by teachers influences their received friendship nominations (Hypothesis 4).

Interaction effects between dyadic teacher (dis)liking and reputational teacher (dis)liking ego were included to examine whether students befriend a peer whom they perceived as (dis)liked by teachers could be moderated by their own reputations regarding teacher (dis)liking. Two separate models were tested: one with interactions between dyadic teacher (dis)liking and reputational teacher liking ego, and another with dyadic teacher (dis)liking and reputational teacher disliking ego.

This study included ego and similarity effects for peer (dis)liking and teacher (dis)liking to control for the tendency of students high on these covariates to nominate more friends and to befriend classmates who are similar on these covariates, respectively. In addition, gender ego, gender alter, and same gender were included as control variables in the analyses.

#### Meta-analytic Procedure

The model was estimated separately for each classroom using the Methods of Moments estimator. The results for each classroom were then combined using a meta-analytic procedure with the metafor package in R (Viechtbauer, 2010). Only networks for which the parameter estimates converged were included in the meta-analysis. The convergence criterion used for the analyses was an overall maximum convergence ratio of less than 0.25, and for all individual parameters, t-ratios for convergence of less than 0.1 in absolute value (Ripley et al., 2023). Appendix 2 provides the individual, friendship, and classroom information.

#### Goodness of Fit

A goodness-of-fit analysis was conducted for each class to assess whether the selected model specification is a good representation of the observed data (Snijders & Steglich, 2015). A non-significant *p*-value indicates that the estimated model does not deviate from the observed data and represents that particular parameter well. Overall, the results for the friendship networks indicated a good representation of the indegree in all classrooms (*p*-values between 0.08 and 0.90).

#### Additional Analysis

To disentangle the direction of the friendship formation effects, the effects were decomposed into a creation and an endowment (also called maintenance) function. Taking the dyadic and reputational teacher liking as an example, for the dyadic effect, a creation function tested whether a new friendship tie would be formed between A and B if A thought the teacher liked B at T1, and an endowment function tested whether A was more likely to continue naming B as a friend if A thought the teacher liked B at T1. For the alter effect, a creation function tested whether A would receive a new friendship tie if A had a high teacher liking reputation, and an endowment function tested whether A was more likely to continue receiving friendship ties if A had a high teacher liking reputation. Appendix 3 shows the results of model with creation and endowment parameters.

## Results

### Descriptive Statistics

Table 1 presents descriptive statistics of the friendship network. The average number of given friendship nominations (outdegree) across the two waves was 3.51 and 3.35, respectively. For the change in friendship ties between T1 and T2, the average Jaccard index between T1 and T2 was 0.36, indicating sufficient stability in the friendship network for social network analysis (Veenstra & Steglich, 2012). As for the number of ties in terms of peer liking, peer disliking, teacher liking, and teacher disliking, on average, students nominated 3.49 of their peers as liked, 2.75 as disliked, 3.74 as liked by teachers, and 2.56 as disliked by teachers at T1. The information for each class is included in Appendix 2.

**Table 1:** Descriptive statistics of friendship networks

	Class size	Density	Reciprocity	Transitivity	Degree	%girl
T1	57.96	0.06	0.51	0.31	3.51	0.48
T2	58.42	0.06	0.53	0.32	3.35	0.48
T1-T2	Jaccard index	Hamming distance	Friendship tie 0 = > 1	Friendship tie 1 = > 0	Friendship tie 1 = > 1	
	0.35	191	93	102	99	

Hamming distance is the number of tie changes; Jaccard index is the proportion of stable ties among the total number of created, dissolved, and stable ties; Friendship tie 0 = > 1 is the number of created friendship ties; Friendship tie 1 = > 0 is the number of dissolved friendship ties; Friendship tie 1 = > 1 is the number of maintained friendship ties

Table 2 displays the descriptive statistics and the correlations of peer (dis)liking and teacher (dis)liking reputations. These reputations show a positively skewed distribution, with most students receiving few or no nominations for peer or teacher (dis)liking, while only a few students receive many. The correlations in Table 2 indicate that reputational peer liking is strongly positively correlated with reputational teacher liking (*r* = 0.59, *p* < 0.001) and weakly negatively correlated with both reputational peer disliking (*r* = –0.26, *p* < 0.001) and reputational teacher disliking (*r* = –0.20, *p* < 0.001). Reputational peer disliking is weakly negatively correlated with reputational teacher liking (*r* = –0.12, *p* < 0.001) and strongly positively correlated with reputational teacher disliking (*r* = 0.75, *p* < 0.001). Additionally, reputational teacher liking is slightly negatively correlated with reputational teacher disliking (*r* = –0.15, *p* < 0.001).

**Table 2:** Descriptive statistics and correlations of the reputation for peer and perceived teacher (dis)liking

	M (SD)	Skewness	Range	1	2	3
1. Rep. peer liking	0.07 (0.06)	1.84	0–0.55	1		
2. Rep. peer disliking	0.05 (0.09)	3.36	0–0.85	−0.26^***^	1	
3. Rep. teacher liking	0.07 (0.16)	3.18	0–1	0.59^***^	−0.12^***^	1
4. Rep. teacher disliking	0.05 (0.12)	3.56	0–0.83	−0.20^***^	0.75^***^	−0.15^***^

*N* = 2544; Rep. refers to Reputational; ^***^p < 0.001

Given the relatively high correlations between peer liking and teacher liking, as well as peer disliking and teacher disliking, attention was focused on potential multicollinearity issues. High correlations (typically above 0.80 or 0.90) can indicate multicollinearity (Tabachnick *&* Fidell, 2007). The highest correlation observed in this study was between teacher disliking and peer disliking reputations (*r* = 0.75), suggesting a low likelihood of multicollinearity. Furthermore, the joint distribution of peer (dis)liking and teacher (dis)liking reputations shows that there is significant overlap at the lower values. As teacher (dis)liking reputation increases, the overlap in the number of students decreases, and the distribution of peer and teacher (dis)liking reputation begins to show more variability (see Appendix 4). Consequently, although the correlations were relatively high, higher levels of teacher (dis)liking reputation did not automatically imply higher levels of peer (dis)liking reputation, and multicollinearity was not considered problematic for the analyses in this study. To further clarify these relationships, the effects of peer (dis)liking and teacher (dis)liking on friendship selection were examined separately in Appendix 5. This separation allows for a clearer understanding of how each type of reputation independently influences friendship dynamics.

### Longitudinal Social Network Analysis

Table 3 presents the results of three SAOM meta-analysis models for friendship networks. Model 0 includes the main effects of dyadic and reputational peer and teacher liking/disliking on friendship selection. Model 1 adds the reputational teacher liking ego as a moderator, and Model 2 adds the reputational teacher disliking ego as a moderator. Because the main effects were consistent across all three models, this study reports the main effects from Model 0 and presents the interactions from Model 1 and Model 2 separately.

**Table 3:** SAOM meta-analysis for friendships networks based on peer and perceived teacher (dis)liking

	Model 0			Model 1			Model 2		
	Est	SE	*p*	Est	SE	*p*	Est	SE	*p*
Outdegree (density)	–1.63	0.12	<0.001	–1.55	0.15	<0.001	–1.70	0.13	<0.001
Reciprocity	1.73	0.04	<0.001	1.67	0.05	<0.001	1.72	0.05	<0.001
GWESP I - > K - > J	1.25	0.04	<0.001	1.30	0.05	<0.001	1.21	0.04	<0.001
Indegree - popularity	–0.03	0.01	<0.001	–0.03	0.01	<0.01	–0.03	0.01	<0.01
Outdegree - popularity	–0.14	0.02	<0.001	–0.14	0.02	<0.001	–0.14	0.02	<0.001
Outdegree - activity	–0.11	0.01	<0.001	–0.12	0.01	<0.001	–0.11	0.01	<0.001
Gender alter (1= female)	0.09	0.04	0.02	0.07	0.04	0.14	0.12	0.04	<0.01
Gender ego (1= female)	–0.26	0.05	<0.001	–0.22	0.05	<0.001	–0.29	0.05	<0.001
Same gender	0.78	0.05	<0.001	0.76	0.06	<0.001	0.82	0.06	<0.001
**Peer liking and disliking**									
Dyadic peer liking	0.51	0.04	<0.001	0.49	0.04	<0.001	0.53	0.04	<0.001
Dyadic peer disliking	–0.13	0.07	0.08	–0.11	0.08	0.18	–0.13	0.08	0.10
Rep. peer liking alter	2.82	0.43	<0.001	2.74	0.49	<0.001	2.63	0.47	<0.001
Rep. peer disliking alter	–1.06	0.39	<0.01	–0.84	0.43	0.050	–1.09	0.41	<0.01
Rep. peer liking ego	–1.95	0.38	<0.001	–1.88	0.45	<0.001	–2.17	0.41	<0.001
Rep. peer liking ego * alter	10.08	3.84	<0.01	7.52	4.05	0.06	10.24	3.93	0.01
Rep. peer disliking ego	0.61	0.34	0.07	0.45	0.40	0.26	0.66	0.35	0.06
Rep. peer disliking ego * alter	3.30	2.47	0.18	3.57	2.56	0.16	3.49	2.69	0.19
**Teacher liking and disliking**									
Dyadic teacher liking	0.18	0.05	<0.001	0.23	0.06	<0.001	0.15	0.06	0.01
Dyadic teacher disliking	0.14	0.09	0.10	0.08	0.13	0.53	0.17	0.09	0.047
Rep. teacher liking alter	–0.59	0.15	<0.001	–0.65	0.17	<0.001	–0.56	0.17	<0.001
Rep. teacher disliking alter	–0.51	0.28	0.07	–0.59	0.36	0.10	–0.54	0.28	0.06
Rep. teacher liking ego	–0.20	0.12	0.10	–0.07	0.19	0.72	–0.19	0.14	0.17
Rep. teacher liking ego * alter	2.03	0.38	<0.001	2.26	0.66	<0.001	2.22	0.42	<0.001
Rep. teacher disliking ego	–0.86	0.28	<0.01	–0.73	0.35	0.04	–1.02	0.36	<0.01
Rep. teacher disliking ego * alter	2.07	0.96	0.03	2.52	1.10	0.02	2.66	1.22	0.03
**Moderating role of teacher (dis)liking reputations**									
Rep. teacher liking ego * Dyadic teacher liking				–0.20	0.41	0.62			
Rep. teacher liking ego * Dyadic teacher disliking				1.70	0.67	0.01			
Rep. teacher disliking ego * Dyadic teacher liking							0.96	0.50	0.053
Rep. teacher disliking ego * Dyadic teacher disliking							0.55	0.67	0.41

The converged classes were 38, 29, and 32 in model 0, model 1, and model 2, separately; Rep. is for reputational

*Est.* unstandardized coefficients, *SE* standard error

#### Peer (Dis)liking

In Model 0, for the dyadic effect of peer liking and disliking, there was a significant positive effect of a peer liking tie at T1 to a friendship tie to T2 (Est. = 0.51; *p* < 0.001) and no significant effect of a peer disliking tie on the formation or continuation of a friendship tie, although it was in the hypothesized direction (Est. = –0.13; *p* = 0.08). These results suggest that students befriend peers they like and tend to avoid befriending peers they dislike. For the reputation effect of peer liking and disliking, peers who received more liking nominations from classmates had more friends (Est. = 2.82; *p* < 0.001) and peers who received more disliking nominations had fewer friends (Est. = −1.06; *p* < 0.01). These results were generally consistent with the hypotheses regarding peer liking and disliking at both the dyadic (Hypothesis 1a) and reputational (Hypothesis 2a and Hypothesis 2b) levels.

In addition to the main effects related to the hypotheses, in Model 0, significant ego effects were found for peer liking reputation (Est. = –1.95; *p* < 0.001) and but not for peer disliking reputations (Est. = 0.61; *p* = 0.07), indicating that students with a higher peer liking sent fewer outgoing friendship nominations, but students’ peer disliking did not influence their outgoing friendship nominations. As for similarity effects, this study found a significant similarity effect for peer liking reputation (Est. = 10.08; *p* < 0.01), but not for peer disliking reputation (Est. = 3.30; *p* = 0.18), suggesting that peers tend to befriend those who have similar levels of peer liking but not peer disliking.

#### Perceived Teacher (Dis)liking

In Model 0, for the direct effects of dyadic teacher (dis)liking on friendship, a significant positive effect was found for a perceived teacher liking tie on a friendship tie (Est. = 0.18; *p* < 0.001), whereas no significant effect was found for a perceived teacher disliking tie on a friendship tie (Est. = 0.14; *p* = 0.10). Regarding the direct effects of reputational teacher (dis)liking on friendship, it was found that peers with a high teacher liking reputation (Est. = –0.59; *p* < 0.001) had fewer friends, confirming the “teachers’ pet” phenomenon. Additionally, peers with a high teacher disliking reputation also tended to have fewer friends, but this effect was not significant (Est. = –0.51; *p* = 0.07). These results were consistent with the hypotheses regarding perceived teacher liking at the dyadic level (Hypothesis 3a), but not with the hypotheses regarding perceived teacher disliking at the dyadic (Hypothesis 3b) and reputational levels (Hypothesis 4).

In terms of interactions, Model 1 found that students with a high reputation for being liked by teachers were more likely to befriend a peer whom they perceived to be disliked by teachers (Est. = 1.70; *p* = 0.01), which surprisingly contradicts Hypothesis 5b. Next, having a high reputation for being liked by teachers did *no*t influence students’ decisions to befriend those whom they believed their teachers liked (Est. = –0.20; *p* = 0.62). Additionally, findings of Model 2 demonstrate that whether students befriended a peer whom they believed their teachers liked or disliked did not depend on their own teacher disliking reputations (Est. = 0.96; *p* = 0.053; Est. = 0.55; *p* = 0.41).

Regarding ego effects in Model 0, adolescents with stronger teacher disliking reputations send fewer friendship nominations than those with weaker disliking reputations (Est. = −0.86; *p* < 0.01). However, adolescents’ teacher liking reputations did not significantly influence their outgoing friendship nominations (Est. = −0.20; *p* = 0.10). For similarity effects, students chose friends with similar levels of teacher liking or disliking reputations (Est. = 2.03; *p* < 0.001; Est. = 2.07; *p* < 0.05). This suggests that friendships may form both among students with a reputation for being liked by teachers and among those with a reputation for being disliked by teachers.

#### Structural Network Effects and Gender

In Model 0, friendships were likely to be reciprocal (Est. = 1.73; *p* < 0.001), and friends of friends tended to be friends (Est. = 1.25; *p* < 0.001). This study found a tendency toward same-gender friendships (Est. = 0.78; *p* < 0.001). Girls sent fewer friendship nominations and received more friendship nominations (Est. = −0.26; *p* < 0.001; Est. = 0.09; *p* < 0.018). The negative indegree-popularity effect (Est. = −0.03; *p* < 0.01) indicated that students who received many nominations received fewer friendship nominations over time. The negative outdegree-popularity effect (Est. = −0.14; *p* < 0.001) and the negative outdegree-activity effect (Est. = −0.11; *p* < 0.001) indicated that students who sent many nominations received and sent fewer friendship nominations over time.

## Discussion

Although previous studies have shown that peer and teacher preferences are associated with adolescents’ peer relationships (e.g., Hendrickx et al., 2017a; Kisfalusi et al., 2022), how peer and teacher (dis)liking influences adolescents’ friendship networks is not fully understood. Therefore, this study used longitudinal social network analysis to examine the role of both dyadic and reputational peer and teacher (dis)liking on friendship selection in a sample of Chinese middle school students. This study also examined whether students’ decisions to befriend a peer whom they perceived as liked or disliked by teachers were moderated by their own teacher (dis)liking reputations. The results of peer (dis)liking indicated that Chinese students tended to form friendships with peers they liked and to avoid those they disliked, although the latter effect was only marginally significant. Moreover, Chinese students with a high peer liking reputation received more friendship nominations, while those with a high peer disliking reputation received fewer. Regarding teacher (dis)liking, Chinese students tended to befriend peers whom they believed their teachers liked but avoided those who were widely perceived to be liked by teachers. Additionally, students with a high teacher liking reputation were more likely to befriend peers they perceived as disliked by teachers. However, perceived teacher disliking had a limited effect on friendship selection at both the dyadic and reputational levels.

### Effects of Peer (Dis)Liking on Friendship dynamics

As expected, Chinese students tended to form friendships with peers they liked and to avoid those they disliked, although the latter effect was only marginally significant. This is consistent with widely accepted definitions of peer liking, peer disliking, and friendship. Liking involves positive feelings and attitudes toward others, whereas disliking involves negative feelings and attitudes (Buhs & Ladd, 2001; Card, 2010; Hughes & Im, 2016). Friendship, however, is based on mutual attraction and is characterized by emotional support and spending time together (Hartup & Stevens, 1997; Wagner, 2019). Thus, when peers like certain classmates, they tend to befriend them; when peers dislike certain classmates, they tend to reject them by ignoring, ridiculing, or excluding them from activities (Buhs & Ladd, 2001).

Consistent with the hypotheses regarding peer liking and disliking reputations (Hypothesis 2a and Hypothesis 2b), Chinese students who were liked by many others (i.e., those with a peer liking reputation) received more friendship nominations, and students who were disliked by others (i.e., those with a peer disliking reputation) received fewer friendship nominations. Being liked or disliked by many others in the classroom indicated higher or lower social status among peers. Students with higher social status tend to be more attractive as potential friends, while those with lower status are often avoided.

Another interesting finding relates to the effect of peer liking reputation on friendship selection. Specifically, in line with previous research on popularity (Palacios et al., 2022), students with high peer liking reputations tend to receive more but send fewer friendship nominations, and they befriend only those who also have high peer liking reputations. This is consistent with previous findings on popularity in a sample of Chilean and European adolescents (Dijkstra et al., 2013; Palacios et al., 2022), suggesting that students with high social status (well-liked or popular) are more selective in their friendship nominations in both Chinese and Western adolescents samples.

### Effects of Teacher (Dis)Liking on Friendship dynamics

#### Dyadic and Reputational Teacher Liking and Friendship Selection

This study found that Chinese students tended to befriend those whom they perceived to be liked by teachers, but tended to avoid befriending those with high teacher liking reputations (i.e., those who were widely perceived as liked by teachers). This finding suggests the dual nature of the effects of perceived teacher liking on friendship dynamics in Chinese middle schools. On the one hand, from a dyadic perspective, teachers act as a social referent; students tend to like and befriend peers they believe are liked by teachers, consistent with previous work among Dutch adolescents (Hendrickx et al., 2017a). On the other hand, from a reputational perspective, students who are widely perceived as being liked by teachers may be labeled as a teacher’s pet (Babad, 2009). This label can carry a negative connotation, leading peers to avoid forming friendships with those students. Students who view the teacher’s favoritism as unfair may avoid forming friendships with these “teacher’s pets,” suggesting a potential social cost associated with being perceived as favored by teachers. The teacher’s pet phenomenon is common in Chinese education because Chinese culture emphasizes valuing and respecting teachers as authorities, promoting obedience and conflict avoidance (Chan & Chan, 2005; Jia et al., 2009). Many Chinese students view their teachers as role models, so perceiving teacher favoritism as unfair may lead to disappointment in teachers and result in dislike for the favored students.

The different findings regarding dyadic and reputational teacher liking on friendship selection suggest that the classmates whom students believed their teachers liked from a dyadic perspective may not necessarily match those with a high teacher liking reputation. In this study, teacher liking reputation scores were skewed, with most students receiving few or no perceived teacher liking nominations, while only a few received many such nominations. This disparity indicates that students with a high teacher liking reputation represent a small subset of the overall peer network, reflecting the collective perceptions of classmates regarding teachers’ attitudes toward peers (Hughes & Im, 2016). This aligns with previous work suggesting that, by definition, only a few students in a classroom are considered “teachers’ pet” (Babad, 2009).

Another important finding of this study is the relatively high correlation between peer liking and teacher liking reputations. However, distinct patterns of friendship selection emerged: students with high peer liking reputations received more friendship nominations, while those with high teacher liking reputations received fewer. Both the high correlation and the “teacher’s pet” effect are consistent with prior studies indicating that a teacher’s liking reputation can positively predict both peer liking and peer disliking reputation among Dutch adolescents (Hendrickx et al., 2017b). These high correlations suggest that students who are well-liked by their peers are generally perceived as well-liked by their teachers; however, the variance in the correlation suggests that peer and teacher perceptions do not always align in all students, which is also supported by the joint distribution table of peer liking and teacher liking reputations in this study. This discrepancy implies that there is a group of students who are perceived to be well-liked by teachers, but not by peers, and thus, may be labeled as “teachers’ pets”. This label may cause peers to avoid forming friendships with these students.

The effects of dyadic perceived teacher liking and friendship selection were found to depend on students’ teacher liking reputations. Contrary to expectations, students with a teacher liking reputation befriended rather than avoided peers whom they believed teachers disliked, revealing an unexpected pattern in the students’ friendship selection behavior. A potential explanation is that these students with a teacher liking reputation, who are often seen as a “teachers’ pets”, were aware of their situation in the classroom. This study found that students with high a teacher liking reputation were avoided by others as friends and withdrew from nominating others as friends. They may have tried to shed the “teachers’ pet” label by befriending peers whom they believed teachers disliked. It may also be that these children did not have many friendship options and hence, via default selection, ended up with peers being disliked by teachers as friends. Another explanation is that, due to the skewed distribution of teacher liking reputations, only a few students had a high teacher liking reputation while most students did not. Compared to themselves, students with a reputation for being liked by the teacher may have been biased to believe that most other students in the class were disliked by the teacher. As a result, their friends are more likely to be students whom they believe the teacher dislikes.

#### Dyadic and Reputational Teacher Disliking and Friendship Selection

This study found no significant association between teacher disliking and friendship selection at the dyadic and reputational levels, with the dyadic-level association being only marginally significant when moderated by teacher disliking reputation. This suggests a limited role for teacher disliking in shaping the friendship network of Chinese adolescents. One possible explanation is that while Chinese teachers may dislike students with poor academic performance or behavior (e.g., aggression), these student characteristics may not be critical factors in friendship formation. For example, aggressive children are usually disliked by teachers (e.g., Chang, 2003), but some may still be popular among their peers and attractive to be friends with (Cillessen & Mayeux, 2004). Furthermore, adolescents often avoid befriending aggressive peers based on their own perceptions rather than peer reputation (Palacios et al., 2022).

Supporting this notion, this study found that students with high teacher disliking reputations tend to send fewer friendship nominations and tend to befriend others who also have high teacher disliking reputations. This suggests that, despite withdrawing from friendship nominations, they still maintain friendships. In addition, students with high teacher disliking reputations tend to befriend peers they perceived as liked by the teacher (although this was marginally significant). This suggests that students who are aware of their negative reputations may attempt to improve their social standing by associating with peers whom they believe the teacher liked. Although teacher disliking reputation may reflect poor teacher-student relationships (e.g., conflict) and increase students’ risk of being victimized (Chang et al., 2007) or disliked (Hendrickx et al., 2017b), their direct influence on friendship selection appears to be nuanced.

The study also uncovered different friendship selection patterns for students with high reputations for peer and teacher disliking, although they were relatively high correlated. Particularly, those with high peer disliking reputations received fewer friendship nominations, while students with high teacher disliking reputations tended to nominate fewer peers and primarily befriended others with similar reputations. These findings suggest that the factors influencing friendship selection differ for peer and teacher disliking reputations. For students with high peer disliking reputations, negative perceptions from classmates significantly impede their ability to form new friendships, leading to social isolation. In contrast, students with high teacher disliking reputations may rely on their social networks or personal qualities to maintain friendships within specific peer groups.

### Strengths, Limitations, and Implications

This study has several strengths. First, it uniquely examines both peer and teacher (dis)liking in friendship dynamics, building on previous research examining the impact of teacher preferences on peer relationships. Second, it distinguishes between the effects of dyadic and reputational perceptions of peer and teacher preferences on adolescents’ friendship selection, thereby advancing the understanding of friendship dynamics within classroom networks. Third, this study examines whether both positive and negative teacher-student relationships moderate how students follow teachers’ preferences when choosing friends, extending previous research to include negative teacher-student relationships. Fourth, by focusing on a sample of Chinese students, the study facilitates cross-cultural comparisons with friendship network studies in Western cultures. The final standout features of this study is its use of a longitudinal social network approach, where individual decisions to change friendships across time points were predicted by network characteristics and individual attributes. By emphasizing the reputational and dyadic effects, this approach provides valuable insights into how friendship dynamics in classroom settings.

Despite these strengths, there are several limitations. First, dyadic and reputational liking and disliking of peers and teachers were treated as constant and exogenous variables. However, research has provided evidence that friendships can predict peer status (Labun et al., 2016) and that peer liking and disliking networks can influence teacher liking and disliking networks (Hendrickx et al., 2017a). Therefore, it would be valuable for future study designs to include these bidirectional associations and co-development dynamics between peer liking and disliking, friendship dynamics, and teacher liking and disliking networks.

Second, this study examined the effects of peer (dis)liking and teacher (dis)liking on friendships separately. However, it is possible that students perceive teachers as liking someone, but students themselves dislike that particular peer. Future studies could include interaction effects to examine how peer conflict and teacher (dis)liking influence friendship networks, or mediation effects to examine how teacher (dis)liking influence peer (dis)liking and then lead to friendship formation or maintenance.

Third, this study did not consider the teachers’ gender, which may influence how peers perceive the teachers’ preferences and fairness. This highlights the need for further research on the role of teachers and peers in adolescent friendship dynamics.

This study has important implications for middle school education. It highlights the importance of teachers being aware of their biases or preferences, as they can serve as referents for students in the classroom. First, teachers can use this mechanism by strategically interacting with a student in a positive way, which can improve how other peers perceive the student (McAuliffe et al., 2009). This approach can help foster positive relationships with students. Second, teachers should avoid creating negative perceptions of “teacher pets”. It is important for teachers to be aware of their interactions with all students and to ensure fair and equitable treatment (Marucci et al., 2021). In addition, teachers should be able to create a positive classroom environment where all students feel valued and respected to mitigate the negative effects of the “teacher’s pet” phenomenon. These strategies are not only relevant in the Chinese educational context, with its unique challenges such as large class sizes, but also applicable internationally, where the dynamics of teacher-student relationships and peer influences play a significant role in educational outcomes. By fostering an inclusive and equitable classroom environment, teachers can positively influence students’ academic adjustment and social development across various cultural and educational settings.

## Conclusion

Despite extensive research on adolescent friendships, there remains a limited understanding of the effects of peer and teacher preferences in shaping friendship selection among Chinese adolescents. Using social network analysis, this study found that peer liking and perceived teacher liking, but not disliking, influenced Chinese adolescents’ friendship choices. Specifically, disliking played a minimal role, as only a reputation for being disliked by peers was associated with fewer friendship nominations. Conversely, liking emerged as a crucial factor: students formed friendships with peers they liked and believed their teachers liked. Interestingly, students who were well-liked by peers tended to have more friendships, whereas those who were widely perceived as liked by teachers had fewer friendships. In addition, students who were widely perceived as liked by teachers were more likely to befriend peers whom they believed teachers disliked. Consequently, these findings underscore the central role of peer and perceived teacher preference in shaping friendship dynamics among Chinese middle school students, suggesting that while positive peer relationships foster social connections, a reputation as a teacher’s favorite may hinder this process.

## Supplementary information

Online supporting information
