# Supplementary material for: The Dominance of Liking: Uncovering Dyadic and Reputational Effects of Peer and Perceived Teacher Likes and Dislikes on Friendship Dynamics Among Chinese Adolescents
Source: J Youth Adolesc. 2024 Oct 30;54(4):903–16. doi: 10.1007/s10964-024-02104-5 (PMC11933165; doi:10.1007/s10964-024-02104-5)
Supplement: Supplementary file 1 — Online supporting information [file 10964_2024_2104_MOESM1_ESM.docx]

**Online supporting information**

**The Dominance of Liking: Uncovering Dyadic and Reputational Effects of Peer and Perceived Teacher Likes and Dislikes on Friendship Dynamics Among Chinese Adolescents**

**Appendices**

**Table of content**

Appendix 1: Parameters of the RSiena model (Table S1).

Appendix 2: Individual, friendship, and classroom information (Table S2 and Table S3).

Appendix 3: Additional analysis examining creation and maintenance effect of peer and teacher (dis)liking (Table S4).

Appendix 4: Joint distributions of peer (dis)liking and teacher (dis)liking nominations (Table S5).

Appendix 5: Additional analysis examining the role of peer and teacher (dis)liking separately (Table S6).

**Appendix 1. Parameters of the RSiena** **model**

Table S1 Explanation of the Parameters in the RSiena Model

| Parameter | Effect name in Rsiena | Explanation |
| --- | --- | --- |
| *Structural effects* |  |  |
| Rate function |  | The average frequency with which actors get the opportunity to change a friendship tie in the simulation process |
| Outdegree | density | The basic tendency to form friendships |
| Reciprocity | recip | The tendency to reciprocate friendships |
| Transitivity | gwespFF | Tendency to send friendship ties that are embedded in transitive triads |
| Transitivity | gwespBB | Tendency to send friendship ties that are embedded in transitive triads |
| Indegree-popularity | inpop | Tendency to send friendship ties to adolescents with higher indegree |
| Outdegree-activity | outact | Tendency of adolescents with higher outdegree to send more friendship ties |
| Outdegree-popularity | outpop | Tendency to send friendship ties to adolescents with higher outdegree |
| *Daydic effects* |  |  |
| W→ X | X | Effect of a tie in network W on a tie in network X (for the same dyad i → j) |
| *Covariates effects* |  |  |
| Ego effect | egoX | Tendency of adolescents with high attribute levels (e.g., peer liking reputation) to send more friendship ties |
| Alter effect | altX | Tendency of adolescents with high attribute levels (e.g., peer liking reputation) to receive more friendship ties |
| Ego * Alter | egoXaltX | Interaction of the above two: Tendency of adolescents with high attribute levels to send friendship ties to others with high attribute levels |
| Girl ego | egoX | Tendency of girls to send friendship ties |
| Girl alter | altX | Tendency of girls to receive friendship ties |
| Same gender | sameX | Tendency to send friendship ties toward same-gender peers |
| Interaction |  |  |
| Ego * W→ X | egoX*X | Interaction of ego effect and daydic effect, tendency of adolescents with high attribute levels in network W send to send a tie in network X |

**Appendix 2. Individual, friendship, and classroom information**

Table S2 Overview of Individual and Classroom Information

| Class | size | density T1 | reciprocity T1 | transitivity T1 | cycle T1 | % girl T1 | density T2 | reciprocity T2 | transitivity T2 | cycle T2 | % girl T2 |
| --- | --- | --- | --- | --- | --- | --- | --- | --- | --- | --- | --- |
| 1 | 51 | .08 | .57 | .36 | .29 | .51 | .08 | .54 | .45 | .33 | .51 |
| 2 | 50 | .08 | .52 | .31 | .25 | .48 | .07 | .54 | .34 | .26 | .48 |
| 3 | 50 | .07 | .49 | .32 | .23 | .50 | .07 | .53 | .36 | .28 | .49 |
| 4 | 49 | .08 | .50 | .31 | .23 | .48 | .08 | .52 | .33 | .24 | .48 |
| 5 | 49 | .07 | .50 | .36 | .26 | .44 | .07 | .56 | .35 | .27 | .46 |
| 6 | 49 | .07 | .49 | .31 | .23 | .51 | .08 | .47 | .32 | .21 | .51 |
| 7 | 62 | .05 | .53 | .39 | .32 | .36 | .05 | .50 | .34 | .22 | .36 |
| 8 | 68 | .05 | .59 | .39 | .32 | .48 | .05 | .63 | .43 | .38 | .48 |
| 9 | 62 | .05 | .51 | .30 | .20 | .32 | .05 | .51 | .33 | .26 | .32 |
| 10 | 65 | .06 | .56 | .38 | .30 | .48 | .05 | .55 | .36 | .30 | .47 |
| 11 | 61 | .06 | .46 | .35 | .26 | .36 | .05 | .52 | .35 | .27 | .36 |
| 12 | 65 | .05 | .48 | .32 | .22 | .47 | .05 | .58 | .25 | .19 | .47 |
| 13 | 60 | .05 | .52 | .39 | .28 | .34 | .05 | .43 | .41 | .24 | .36 |
| 14 | 57 | .07 | .63 | .51 | .44 | .64 | .06 | .56 | .39 | .30 | .64 |
| 15 | 66 | .04 | .48 | .43 | .29 | .40 | .04 | .52 | .40 | .29 | .40 |
| 16 | 53 | .08 | .55 | .36 | .27 | .68 | .07 | .58 | .40 | .32 | .68 |
| 17 | 55 | .06 | .46 | .26 | .17 | .64 | .06 | .55 | .33 | .26 | .65 |
| 18 | 63 | .06 | .45 | .29 | .21 | .47 | .05 | .52 | .27 | .18 | .47 |
| 19 | 59 | .07 | .52 | .25 | .19 | .63 | .06 | .55 | .33 | .23 | .63 |
| 20 | 60 | .06 | .46 | .25 | .21 | .39 | .05 | .45 | .25 | .20 | .39 |
| 21 | 49 | .08 | .51 | .34 | .26 | .45 | .07 | .57 | .33 | .31 | .46 |
| 22 | 49 | .07 | .51 | .40 | .29 | .45 | .07 | .67 | .47 | .42 | .45 |
| 23 | 49 | .07 | .45 | .38 | .24 | .47 | .07 | .53 | .40 | .30 | .46 |
| 24 | 50 | .07 | .52 | .30 | .25 | .47 | .07 | .57 | .33 | .28 | .48 |
| 25 | 50 | .06 | .45 | .27 | .21 | .48 | .06 | .44 | .39 | .25 | .48 |
| 26 | 49 | .07 | .42 | .36 | .22 | .48 | .06 | .47 | .33 | .22 | .50 |
| 27 | 64 | .06 | .52 | .25 | .20 | .52 | .06 | .49 | .23 | .15 | .53 |
| 28 | 67 | .06 | .45 | .22 | .15 | .51 | .06 | .53 | .30 | .24 | .51 |
| 29 | 64 | .07 | .59 | .22 | .16 | .48 | .06 | .59 | .26 | .22 | .48 |
| 30 | 65 | .06 | .51 | .30 | .23 | .49 | .05 | .59 | .26 | .22 | .49 |
| 31 | 62 | .06 | .54 | .28 | .22 | .48 | .06 | .50 | .28 | .23 | .48 |
| 32 | 66 | .05 | .50 | .22 | .15 | .52 | .05 | .49 | .25 | .16 | .52 |
| 33 | 63 | .05 | .50 | .22 | .18 | .49 | .05 | .53 | .21 | .16 | .48 |
| 34 | 65 | .06 | .53 | .28 | .24 | .46 | .06 | .52 | .30 | .23 | .47 |
| 35 | 65 | .04 | .43 | .26 | .15 | .47 | .05 | .49 | .26 | .19 | .47 |
| 36 | 62 | .06 | .54 | .22 | .17 | .45 | .05 | .48 | .21 | .16 | .45 |
| 37 | 65 | .06 | .54 | .31 | .25 | .46 | .06 | .56 | .33 | .25 | .46 |
| 38 | 64 | .06 | .47 | .30 | .22 | .51 | .05 | .46 | .23 | .18 | .49 |
| 39 | 66 | .06 | .47 | .17 | .11 | .50 | .05 | .53 | .25 | .19 | .50 |
| 40 | 64 | .07 | .48 | .21 | .13 | .47 | .06 | .47 | .25 | .17 | .48 |
| 41 | 65 | .05 | .52 | .21 | .19 | .50 | .05 | .53 | .26 | .21 | .50 |
| 42 | 48 | .07 | .57 | .29 | .24 | .52 | .08 | .58 | .32 | .25 | .52 |
| 43 | 48 | .08 | .57 | .29 | .23 | .52 | .07 | .65 | .39 | .35 | .51 |
| 44 | 47 | .07 | .57 | .42 | .35 | .53 | .07 | .53 | .30 | .20 | .53 |
| 45 | 48 | .09 | .54 | .29 | .24 | .52 | .08 | .54 | .32 | .22 | .51 |
| Average | 57 | .06 | .51 | .31 | .23 | .48 | .06 | .53 | .32 | .24 | .48 |

*Notes*. a Density was calculated as N of ties divided by the total number of ties.

b Reciprocity was calculated as 2M/(2M + A), where M = mutual ties and A = asymmetric ties.

c Hamming distance is the number of tie changes.

d Jaccard index is the fraction of stable ties relative to all new, lost, and stable ties.

Sample contains complete data from all respondents.

Table S3 Changes in Friendship Networks Variables across Two Observations in All Classrooms (*N* = 45)

| Class | Fr 0 => 0 | Fr 0 => 1 | Fr 1 => 0 | Fr 1 => 1 | Distance | Jaccard | Missing |  |
| --- | --- | --- | --- | --- | --- | --- | --- | --- |
| 1 | 2219 | 79 | 84 | 118 | 163 | .42 | .02 |  |
| 2 | 2101 | 71 | 91 | 89 | 162 | .36 | .04 |  |
| 3 | 2248 | 80 | 71 | 102 | 144 | .40 | .02 |  |
| 4 | 2048 | 69 | 74 | 113 | 143 | .44 | .02 |  |
| 5 | 2157 | 73 | 83 | 88 | 149 | .36 | .02 |  |
| 6 | 1995 | 98 | 73 | 90 | 171 | .35 | .04 |  |
| 7 | 3398 | 84 | 93 | 85 | 167 | .32 | .12 |  |
| 8 | 4007 | 108 | 126 | 114 | 224 | .33 | .04 |  |
| 9 | 3212 | 74 | 96 | 94 | 170 | .36 | .11 |  |
| 10 | 3895 | 86 | 128 | 119 | 212 | .36 | .04 |  |
| 11 | 3491 | 93 | 104 | 95 | 190 | .33 | .03 |  |
| 12 | 3900 | 109 | 113 | 110 | 214 | .33 | .07 |  |
| 13 | 3137 | 101 | 124 | 60 | 202 | .21 | .10 |  |
| 14 | 3027 | 72 | 104 | 103 | 176 | .37 | .00 |  |
| 15 | 3899 | 79 | 105 | 77 | 184 | .30 | .06 |  |
| 16 | 2783 | 91 | 76 | 130 | 155 | .44 | .00 |  |
| 17 | 2657 | 75 | 83 | 101 | 158 | .39 | .02 |  |
| 18 | 3130 | 103 | 105 | 79 | 201 | .28 | .13 |  |
| 19 | 3102 | 96 | 100 | 124 | 196 | .39 | .00 |  |
| 20 | 2850 | 89 | 120 | 68 | 209 | .25 | .12 |  |
| 21 | 2104 | 67 | 79 | 102 | 138 | .41 | .04 |  |
| 22 | 1982 | 65 | 66 | 95 | 131 | .42 | .06 |  |
| 23 | 2083 | 56 | 63 | 102 | 114 | .46 | .02 |  |
| 24 | 2293 | 76 | 90 | 91 | 158 | .35 | .00 |  |
| 25 | 2138 | 69 | 72 | 73 | 141 | .34 | .04 |  |
| 26 | 2220 | 69 | 89 | 72 | 151 | .31 | .04 |  |
| 27 | 3851 | 119 | 127 | 130 | 232 | .35 | .01 |  |
| 28 | 4050 | 107 | 113 | 152 | 220 | .41 | .00 |  |
| 29 | 3657 | 105 | 126 | 144 | 231 | .38 | .00 |  |
| 30 | 3619 | 124 | 129 | 96 | 253 | .28 | .05 |  |
| 31 | 3326 | 103 | 124 | 107 | 227 | .32 | .03 |  |
| 32 | 3825 | 114 | 118 | 103 | 232 | .31 | .03 |  |
| 33 | 3783 | 109 | 115 | 91 | 208 | .29 | .01 |  |
| 34 | 3744 | 125 | 116 | 111 | 236 | .32 | .05 |  |
| 35 | 3491 | 109 | 101 | 75 | 210 | .26 | .09 |  |
| 36 | 3581 | 111 | 113 | 101 | 211 | .31 | .03 |  |
| 37 | 3604 | 113 | 124 | 127 | 237 | .35 | .05 |  |
| 38 | 3740 | 114 | 142 | 101 | 243 | .28 | .04 |  |
| 39 | 3904 | 132 | 155 | 99 | 287 | .26 | .00 |  |
| 40 | 3782 | 115 | 147 | 116 | 260 | .31 | .00 |  |
| 41 | 3950 | 117 | 111 | 112 | 215 | .33 | .03 |  |
| 42 | 1999 | 93 | 75 | 89 | 168 | .35 | .00 |  |
| 43 | 2056 | 78 | 91 | 80 | 164 | .32 | .02 |  |
| 44 | 1930 | 82 | 81 | 69 | 163 | .30 | .00 |  |
| 45 | 2067 | 93 | 103 | 89 | 189 | .31 | .00 |  |
| Average | 3023 | 93 | 102 | 99 | 191 | .34 | .04 |  |

**Appendix 3. Additional analysis examining creation and maintenance effect of peer and teacher (dis)liking**

**Peer (Dis)liking**. Table S4 presents the results of the SAOM meta-analysis for friendship networks distinguishing between creation and endowment effects. Estimates and standard errors are based on 19 classrooms. For the direct effect of peer liking, a positive effect was observed for friendship creation (Est. = 0.42; *p* < .01) and maintenance (Est. = 0.51; *p* < .001), suggesting that a peer liking tie at T1 would lead to the formation and continuation of a friendship tie at T2. Conversely, a negative effect was found for peer disliking on friendship creation (Est. = -0.59; *p* < .05). The effect for peer disliking on friendship maintenance was in the same direction but not significant (Est. = -1.01; *p* = .09). These findings suggest that a peer disliking tie at T1 resulted in either the absence of friendship formation or the dissolution of a friendship tie at T2. For the reputation effect of peer liking, a significant creation effect was observed (Est. = 0.19; *p* < .05), indicating that peers who received more liking nominations from classmates at T1 were more likely to form new friendship ties at T2. However, there were no significant effect of peer liking alter on friendship maintenance (Est. = 0.12; *p* = .19), of peer disliking alter on friendship creation (Est. =-0.03; *p* = .69), or maintenance (Est. =-0.18; *p* = .10).

**Perceived Teacher (Dis)liking**. For the direct effect of teacher liking, a positive effect on friendship creation was found (Est. = 0.59; *p* < .01), indicating that a teacher liking tie at T1 would lead to the formation of a new friendship tie at T2. However, the reputation effect of teacher liking alter on friendship creation was significantly negative (Est. = -0.16; *p* < .001), indicating that peers with a teacher liking reputation at T1 received fewer new friendship ties at T2. Furthermore, no significant direct effect was found for teacher liking on friendship maintenance (Est. = 0.11; *p* = .58), teacher disliking on friendship creation (Est. = 0.18; *p* = .41), or teacher disliking on friendship maintenance (Est. = 0.33; *p* = .30). Similarly, no significant reputation effect was observed for teacher liking alter on friendship maintenance (Est. = 0.04; *p* = .32), teacher disliking alter on friendship creation (Est. =-0.09; *p* = .16), or teacher disliking alter on friendship maintenance (Est. =-0.02; *p* = .83).

Table S4 SAOM Meta-analysis for Friendships Networks based on Peer and Perceived Teacher (Dis)Liking

| Effects |  | Est | SE | *p* |
| --- | --- | --- | --- | --- |
| Basic rate parameter friendship |  | 7.74 | 0.40 | <.001 |
| Outdegree (density) |  | -1.40 | 0.19 | <.001 |
| Reciprocity |  | 1.75 | 0.06 | <.001 |
| GWESP I -> K -> J |  | 0.05 | 0.06 | .40 |
| Indegree - popularity |  | -0.02 | 0.01 | .14 |
| Outdegree - popularity |  | -0.16 | 0.03 | <.001 |
| Outdegree - activity |  | -0.13 | 0.01 | <.001 |
| Gender alter (1= female) |  | 0.12 | 0.06 | .07 |
| Gender ego (1= female) |  | -0.22 | 0.08 | <.01 |
| Same gender |  | 0.86 | 0.10 | <.001 |
| **Peer liking and disliking** |  |  |  |  |
| peer liking maintenance |  | 0.51 | 0.11 | <.001 |
| peer liking creation |  | 0.42 | 0.15 | .01 |
| peer disliking maintenance |  | 1.01 | 0.59 | .09 |
| peer disliking creation |  | -0.59 | 0.23 | .01 |
| peer liking alter maintenance |  | 0.12 | 0.09 | .19 |
| peer liking alter creation |  | 0.19 | 0.08 | .02 |
| peer liking ego |  | -0.13 | 0.05 | .01 |
| peer liking ego * alter |  | 0.11 | 0.03 | .002 |
| peer disliking alter maintenance |  | -0.18 | 0.11 | .10 |
| peer disliking alter creation |  | -0.03 | 0.08 | .69 |
| peer disliking ego |  | 0.05 | 0.05 | .23 |
| peer disliking ego * alter |  | 0.01 | 0.05 | .90 |
| **Teacher liking and disliking** |  |  |  |  |
| teacher liking endowment |  | 0.11 | 0.19 | .58 |
| teacher liking creation |  | 0.59 | 0.17 | <.001 |
| teacher disliking maintenance |  | 0.33 | 0.32 | .30 |
| teacher disliking creation |  | 0.18 | 0.22 | .41 |
| teacher liking alter maintenance |  | 0.04 | 0.04 | .32 |
| teacher liking alter creation |  | -0.16 | 0.03 | <.001 |
| teacher liking ego |  | -0.03 | 0.02 | .18 |
| teacher liking ego * alter |  | 0.02 | 0.01 | <.001 |
| teacher disliking alter maintenance |  | -0.02 | 0.09 | .83 |
| teacher disliking alter creation |  | -0.09 | 0.07 | .16 |
| teacher disliking ego |  | -0.05 | 0.04 | .24 |
| teacher disliking ego* alter |  | 0.04 | 0.01 | .01 |

*Notes*. Est. = unstandardized coefficients; *SE* = standard error; There are 19 classes that converge.

**Appendix 4. Distributions of peer and teacher (dis)liking nominations.**

To clearly present the distribution, this study calculated the joint matrix of reputations for peer (dis)liking and teacher (dis)liking by multiplying the proportion scores, which ranged from 0 to 1, by 10 and rounding them to the nearest integer (Niezink, 2018). The results reveal a strong overlap at low reputation levels (0 and 1) for both teacher and peer liking. As the reputation for teacher liking increases, the number of students in this overlap decreases, leading to greater variability in the distribution of both peer and teacher liking reputations. Similarly, the matrix on the right demonstrates the joint distribution of teacher disliking and peer liking reputations, also revealing a strong overlap at low levels (0 and 1). As teacher disliking increases, the overlap declines.

Table S5 The Join Distribution of Reputation for Peer Liking and Teacher Liking

| Teacher liking reputation | Peer liking reputation | | | | | |  | Teacher disliking reputation | Peer disliking reputation | | | | | | | | |
| --- | --- | --- | --- | --- | --- | --- | --- | --- | --- | --- | --- | --- | --- | --- | --- | --- | --- |
|  | 0 | 1 | 2 | 3 | 4 | 6 |  |  | 0 | 1 | 2 | 3 | 4 | 5 | 6 | 7 | 8 |
| 0 | 1186 | 728 | 42 | 1 | 0 | 0 |  | 0 | 1701 | 287 | 42 | 5 | 0 | 0 | 0 | 0 | 0 |
| 1 | 62 | 143 | 34 | 0 | 0 | 0 |  | 1 | 85 | 112 | 34 | 8 | 3 | 0 | 0 | 0 | 0 |
| 2 | 20 | 56 | 31 | 2 | 0 | 0 |  | 2 | 17 | 43 | 28 | 8 | 2 | 0 | 0 | 0 | 0 |
| 3 | 7 | 34 | 19 | 2 | 0 | 0 |  | 3 | 8 | 26 | 15 | 8 | 6 | 2 | 2 | 0 | 0 |
| 4 | 7 | 24 | 17 | 4 | 0 | 0 |  | 4 | 0 | 8 | 12 | 3 | 1 | 2 | 0 | 0 | 0 |
| 5 | 2 | 13 | 13 | 1 | 0 | 0 |  | 5 | 2 | 7 | 7 | 9 | 1 | 3 | 0 | 0 | 0 |
| 6 | 0 | 8 | 17 | 5 | 1 | 0 |  | 6 | 0 | 3 | 3 | 5 | 4 | 1 | 2 | 0 | 0 |
| 7 | 1 | 11 | 9 | 3 | 1 | 0 |  | 7 | 0 | 2 | 2 | 3 | 3 | 3 | 2 | 2 | 0 |
| 8 | 0 | 5 | 5 | 6 | 0 | 1 |  | 8 | 0 | 1 | 0 | 0 | 3 | 4 | 1 | 2 | 1 |
| 9 | 2 | 6 | 4 | 1 | 4 | 0 |  | — |  |  |  |  |  |  |  |  |  |
| 10 | 0 | 1 | 2 | 1 | 2 | 0 |  | — |  |  |  |  |  |  |  |  |  |

**Appendix 5. Additional analysis examining the role of peer and teacher (dis)liking**

Table S5 presents the results of the SAOM meta-analysis for friendship networks based on peer (dis)liking (M1), teacher (dis)liking (M2), and both peer and teacher (dis)liking (M3). M3 is the model which is included in the manuscript.

**Peer (Dis)liking**. In the peer (dis)liking model, which only included the effects friendship network and peer (dis)liking, a significant dyadic peer liking effect was found (Est. = 0.53; *p* < .001), while the dyadic peer disliking effect was not significant (Est. = -0.08; *p* = .19). Additionally, students with a high peer liking reputation received more friendship nominations (Est. = 1.28; *p* < .001), whereas those with a high peer disliking reputation received fewer nominations (Est. = -1.31; *p* < .001). These results were generally consistent with those observed after including both peer and teacher (dis)liking in M3.

**Teacher (Dis)liking**. In the teacher (dis)liking model, which focused solely on the effects of the friendship network and teacher (dis)liking, a significant dyadic teacher liking effect was found (Est. = 0.28; *p* < .001), while the dyadic teacher disliking effect was not significant (Est. = -0.10; *p* = .19). The two effects were generally consistent with those observed in M3, which included both peer and teacher (dis)liking. However, teacher liking reputation did not significantly influence students’ receiving of friendship nominations (Est. = -0.18; *p* = .11), while it was significant in M3 (Est. = -0.59; *p* < .001), though the effect was in the same direction in both models. Additionally, students with a high teacher disliking reputation received fewer nominations (Est. = -1.07; *p* < .001), whereas this effect was a trend in M3 (Est. = -0.51; *p* = .07). These results show some differences from those observed when both peer and teacher (dis)liking were considered together.

Table S6 SAOM Meta-analysis for Friendships Networks based on Peer and Perceived Teacher (Dis)Liking

|  | M1: Peer-(dis)liking | | |  | M2: Teacher-(dis)liking | | |  | M3: Peer and teacher (dis)liking | | |
| --- | --- | --- | --- | --- | --- | --- | --- | --- | --- | --- | --- |
|  | Est | SE | *p* |  | Est | SE | *p* |  | Est | SE | *p* |
| Outdegree (density) | -1.73 | 0.09 | <.001 |  | -1.66 | 0.12 | <.001 |  | -1.63 | 0.12 | <.001 |
| Reciprocity | 1.73 | 0.04 | <.001 |  | 1.79 | 0.04 | <.001 |  | 1.73 | 0.04 | <.001 |
| GWESP I -> K -> J | 1.24 | 0.04 | <.001 |  | 1.31 | 0.04 | <.001 |  | 1.25 | 0.04 | <.001 |
| Indegree - popularity | -0.01 | 0.01 | .12 |  | 0.01 | 0.01 | .14 |  | -0.03 | 0.01 | <.001 |
| Outdegree - popularity | -0.14 | 0.02 | <.001 |  | -0.19 | 0.02 | <.001 |  | -0.14 | 0.02 | <.001 |
| Outdegree - activity | -0.10 | 0.01 | <.001 |  | -0.16 | 0.01 | <.001 |  | -0.11 | 0.01 | <.001 |
| Gender alter (1= female) | 0.10 | 0.04 | <.001 |  | 0.13 | 0.04 | <.001 |  | 0.09 | 0.04 | .02 |
| Gender ego (1= female) | -0.23 | 0.04 | <.001 |  | -0.28 | 0.05 | <.001 |  | -0.26 | 0.05 | <.001 |
| Same gender | 0.77 | 0.05 | <.001 |  | 0.81 | 0.05 | <.001 |  | 0.78 | 0.05 | <.001 |
| **Peer liking and disliking** |  |  |  |  |  |  |  |  |  |  |  |
| Dyadic peer liking | 0.53 | 0.03 | <.001 |  |  |  |  |  | 0.51 | 0.04 | <.001 |
| Dyadic peer disliking | -0.08 | 0.06 | .19 |  |  |  |  |  | -0.13 | 0.07 | .08 |
| Reputational peer liking alter | 1.28 | 0.26 | <.001 |  |  |  |  |  | 2.82 | 0.43 | <.001 |
| Reputational peer disliking alter | -1.31 | 0.11 | <.001 |  |  |  |  |  | -1.06 | 0.39 | <.01 |
| Reputational peer liking ego | -2.50 | 0.28 | <.001 |  |  |  |  |  | -1.95 | 0.38 | <.001 |
| Reputational peer liking ego * alter | 15.15 | 4.18 | <.001 |  |  |  |  |  | 10.08 | 3.84 | <.01 |
| Reputational peer disliking ego | -0.23 | 0.17 | .17 |  |  |  |  |  | 0.61 | 0.34 | .07 |
| Reputational peer disliking ego * alter | 4.17 | 1.39 | .003 |  |  |  |  |  | 3.30 | 2.47 | .18 |
| Teacher liking and disliking |  |  |  |  |  |  |  |  |  |  |  |
| Dyadic teacher liking |  |  |  |  | 0.28 | 0.05 | <.001 |  | 0.18 | 0.05 | <.001 |
| Dyadic teacher disliking |  |  |  |  | 0.10 | 0.09 | .26 |  | 0.14 | 0.09 | .10 |
| Reputational teacher liking alter |  |  |  |  | -0.18 | 0.11 | .11 |  | -0.59 | 0.15 | <.001 |
| Reputational teacher disliking alter |  |  |  |  | -1.07 | 0.18 | <.001 |  | -0.51 | 0.28 | .07 |
| Reputational teacher liking ego |  |  |  |  | -0.54 | 0.10 | <.001 |  | -0.20 | 0.12 | .10 |
| Reputational teacher liking ego * alter |  |  |  |  | 2.74 | 0.46 | <.001 |  | 2.03 | 0.38 | <.001 |
| Reputational teacher disliking ego |  |  |  |  | -0.38 | 0.14 | <.01 |  | -0.86 | 0.28 | <.01 |
| Reputational teacher disliking ego * alter |  |  |  |  | 2.56 | 0.68 | <.001 |  | 2.07 | 0.96 | .03 |

*Notes*. Est. = unstandardized coefficients; *SE* = standard error. The converged classes were 38, 40, and 38, separately. Rep. is for Reputational. ^*^ *p* < .05, ^**^ *p* < .01, ^** *^*p* < .001.

**Reference**

Niezink, N. M. D. (2018). *Modeling the dynamics of networks and continuous behavior*. [Doctoral dissertation]: University of Groningen.
